# Picropodophyllin (PPP) is a potent rhabdomyosarcoma growth inhibitor both in vitro and in vivo

**DOI:** 10.1186/s12885-017-3495-y

**Published:** 2017-08-09

**Authors:** Maciej Tarnowski, Marta Tkacz, Katarzyna Zgutka, Joanna Bujak, Patrycja Kopytko, Andrzej Pawlik

**Affiliations:** Department of Physiology, Pomeranian Medical University, al. Powstańców Wielkopolskich 72, 70-111 Szczecin, Poland

**Keywords:** Rhabdomyosarcoma, IGF1R, Pircropodophyllin, Cancer

## Abstract

**Background:**

Insulin-like growth factors and insulin are important factors promoting cancer growth and metastasis. The molecules act through IGF1 (IGF1R) and insulin (InsR) receptors. Rhambodmyosarcomas (RMS) overproduce IGF2 – a potent ligand for IGF1R and, at the same time, highly express IGF1 receptor. The purpose of the study was to evaluate possible application of picropodophyllin (PPP) – a potent IGF1R inhibitor.

**Methods:**

In our study we used a number of in vitro assays showing influence of IGF1R blockage on RMS cell lines (both ARMS and ERMS) proliferation, migration, adhesion, cell cycling and signal transduction pathways. Additionally, we tested possible concomitant application of PPP with commonly used chemotherapeutics (vincristine, actinomycin-D and cisplatin). Moreover, we performed an in vivo study where PPP was injected intraperitoneally into RMS tumor bearing SCID mice.

**Results:**

We observed that PPP strongly inhibits RMS proliferation, chemotaxis and adhesion. What is more, application of the IGF1R inhibitor attenuates MAPK phosphorylation and cause cell cycle arrest in G2/M phase. PPP increases sensitivity of RMS cell lines to chemotherapy, specifically to vincristine and cisplatin. In our in vivo studies we noted that mice treated with PPP grew smaller tumors and displayed significantly decreased seeding into bone marrow.

**Conclusions:**

The cyclolignan PPP effectively inhibits RMS tumor proliferation and metastasis in vitro and in an animal model.

**Electronic supplementary material:**

The online version of this article (doi:10.1186/s12885-017-3495-y) contains supplementary material, which is available to authorized users.

## Background

Rhabdomyosarcoma (RMS) is one of the most common soft tissue tumours among children. It is derived from embryonic mesenchymal or embryonic cells with the potential to differentiate into the skeletal muscle [1]. There are two major histological types: embryonal (ERMS) and alveolar (ARMS). The embryonic type is more common, nearly 2/3 of cases, and is generally associated with a good prognosis. The alveolar type is less common and in contrast to ERMS, is characterised by a significantly worse prognosis due to aggressive growth and increased metastatic potential [2, 3]. The survival of the patient is highly dependent on the clinical features of the RMS, such as the location of the tumour, the severity of the disease and the treatment [4, 5]. In the case of RMS, as well as many other types of solid tumours, the treatment strategy frequently uses a combination of therapies consisting of surgery, radiation and chemotherapy.

Insulin-like growth factors (IGF1 and IGF2) and insulin (Ins) play an important role in the normal growth and differentiation of skeletal muscle cells and muscle tissue homeostasis in adult life. These factors are especially important for muscle cell proliferation and regeneration. Both IGF1 and IGF2 act through the tyrosine kinase insulin-like factor 1 receptor (IGF1R), which is widely overexpressed in multiple childhood sarcomas, including rhabdomyosarcomas [6–12], and other cancers such as breast cancer, prostate cancer and lung cancer [13–16]. Furthermore, IGF2, the potent ligand of IGF1R, is also overproduced in rhabdomyosarcomas [6–12]. This overactive IGF signalling axis is associated with decreased survival in RMS [12]. Thus, together with its receptor, IGF1 and IGF2 form a very potent axis of autocrine signalling that stimulates the proliferation of RMS tumours.

The oncogenic potential of IGF1R has been repeatedly documented in a large variety of solid tumours [13–16]. IGF1R seems to be a promising target for cancer treatment and several strategies blocking IGF1R activity are undergoing clinical trials [17–19].

Picropodophyllin PPP is a cyclolignan, an epimer of podophyllotoxin (PPT), that occurs naturally and can be isolated from certain plant species. Although the exact mechanism has not been established, PPP has been shown to specifically inhibit IGF1R activity by blocking IGF1R phosphorylation and downstream signalling, such as Akt and extracellular signal-regulated kinase Erk (MAPK) phosphorylation [20, 21]. PPP also induces apoptosis of malignant cells, as well as tumour regression in different tumour models [20–23]. Additionally, PPP interferes with microtubule assembly and, importantly, does not interfere with the highly similar insulin receptor and other tyrosine kinase receptors [22].

In our study, we used two human RMS cell lines, typical for ARMS and ERMS, and a xenotransplantation model of human rhabdomyosarcoma. PPP efficiently blocked in vitro activity of RMS cells, specifically migration and proliferation, and when used in an in vivo model, treatment with PPP lead to a decrease in tumour volume after two weeks and a decrease in the spread of cancer cells to bone marrow.

## Methods

### Cell lines

Two human RMS cell lines RH30 (CRL-2061; ARMS) and RD (CCL-136; ERMS) (ATCC) were used in this study. RMS cells were cultured in RPMI 1640 medium (Sigma Aldrich), supplemented with penicillin, streptomycin (100 IU/ml and 10 μg/ml, respectively) (Life Technologies) and 10% heat-inactivated FBS (fetal bovine serum, Life Technologies). The cell culture was conducted at an initial cell density of 2.5 × 10^4^ cells/flask (Corning) in a humidified atmosphere at 37 °C in 5% CO_2_ and the media were changed every two days.

### Receptor expression analysis by flow cytometry

The expression of IGF1R, InsR in RMS cell lines was evaluated by flow cytometry as previously described [24]. Briefly, the receptor expression was assayed with phycoerythrin (PE)-anti-IGF1R monoclonal antibody Clone 33,255 and anti–human/mouse Insulin R/CD220 conjugated with APC (R&D Systems). The cells were stained, washed and re-suspended in PBS (Ca^2+^- and Mg^2+^-free). Analysis was performed on the Navios flow cytometer (Beckman Coulter).

### Cell cycle analysis

After 72 h of incubation with or without 0.1, 0.5, 1, 2, and 3 μM PPP (Tocris), the cells were collected, washed, centrifuged and resuspended in 1 ml RPMI 1640 medium supplemented with 10% fetal bovine serum at a concentration of 10^6^ cells/ml. 2 μl of Vybrant DyeCycle Orange Stain (Invitrogen) cell permeable DNA dye was added to assess cell cycle stage by flow cytometer.

### Chemotaxis assay

The assay was performed as previously described [24, 25]. Briefly, the 8-μm polycarbonate membranes covered with 50 μL of 0.5% gelatin were used. Cells were detached with 0.5 mmol/l ethylendiaminetetraacetic acid (EDTA), washed and resuspended in RPMI 1640 with 0.5% BSA. The cells were seeded at a density of 3 × 10^4^ in 120 μL into the upper chambers of Transwell inserts (Costar Transwell; Corning Costar). For the PPP-treated, the cells were preincubated with PPP (0.1 μM) for 30 min and then lower chambers were filled with IGF1, IGF2 and INS or 0.5% BSA RPMI 1640 (control) with 0.1 μM PPP. After 24 h, the inserts were removed from the Transwells. Cells remaining in the upper chambers were removed with cotton wool and the transmigrated cells were stained by HEMA 3 (Fisher Scientific) and counted.

### Colony formation assay

The assay was performed as previously described [24].

### Cell proliferation

Cells were plated in culture flasks at an initial density of 10^3^ cells/cm^2^ in the presence or absence of PPP (0.01–1 μM), and selected chemotherapeutics (vincrstine, actinomycin-D, cisplatin; all from Sigma Aldrich). The cell number was counted at 24, 48, and 72 h after culture start. At the time points, cells were trypsinized from the culture plates and the cells were counted using a cytometer (Beckman Coulter).

### Adhesion of RMS cells to fibronectin

In order to make the cells quiescent they were incubated for 4 h with 0.5% BSA in RPMI before stimulation with IGF1 (100 ng/mL), IGF2 (100 ng/mL), or insulin (10 ng/mL) for 5 min with or without PPP (0.1 μM). PPP-treated cells were additionally pretreated for 30 min with 0.1 μM PPP. The protocol was followed accordingly to the [25].

### Western blot visualization of phosphorylation of intracellular pathway proteins

Before the experiment PPP-treated cells were additionally pretreated for 72 h with 0.1 μM PPP. The cells stimulated with following doses of: IGF1 (100 ng/mL), IGF2 (100 ng/mL), and insulin (10 ng/mL) for 5 min. Western blots were performed as previously described [24, 25]. The membranes were developed with an enhanced chemiluminescence (ECL) reagent (GE Healthcare), dried, and visualized by Chemidoc transilluminator (BioRad).

### Annexin V/PI assays for apoptosis

For apoptosis assay, cells were stained with Annexin V–FITC and PI, and checked for apoptosis by flow cytometer accordingly to the manufacturer’s protocol (BD PharMingen) and as previously described [24].

### Animal care and ethics statement

Approval for use of laboratory animals was obtained from Local Ethics Committee for Animal Studies in Szczecin, affiliated at West Pomeranian University of Technology, Szczecin, Poland. Male SCID-Beige inbred mice (Charles River Laboratories, Germany), 4 to 6 weeks old, were used in this study. These animals were housed in pathogen-free conditions and provided with food and water at the facility of Pomeranian Medical University.

### Xenotransplants of RMS cells into immunodeficient mice

In order to evaluate the metastatic behavior of RH30 cells in vivo (6 × 10^6^ per mouse), the cells were inoculated into the hind limb muscles of SCID-Beige inbred mice and the experiment was performed as previously described [25].

### Statistical analysis

The results are presented as mean ± standard error of the mean (SEM). Statistical analysis of the data was performed using the nonparametric Mann-Whitney test or Student t-test, with *p* < 0.05 considered significant.

## Results

### Rhabdomyosarcoma cell lines highly express IGF1R and insulin receptors

**Fig. 1:**
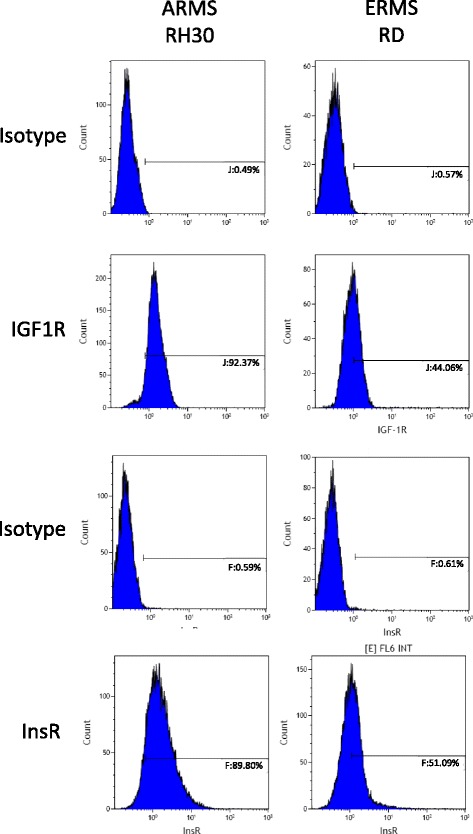
Insulin and insulin-like growth factor receptor expression on RMS cells. The expression of IGF1R, InsR in RMS cell lines was evaluated by flow cytometry analysis. The antigens were detected with anti–human/mouse Insulin R/CD220 conjugated with APC (R&D Systems); phycoerythrin (PE)-anti-IGF1R monoclonal antibody Clone 33,255 (R&D Systems)

IGF1R and insulin receptors (InsR) are commonly overexpressed in human RMS [6–12]. Two cell lines tested in our study, RH30 and RD, which are characteristic representatives of ARMS and ERMS Rhabdomyosarcoma subtypes, highly expressed the two receptors. We observed 92% and 90% of RH30 cells stained positively for IGF1R and InsR, respectively. In case of RD, 44% and 51% cells were positive for IGF1R and InsR, respectively (Fig. 1). Additionally we performed an mRNA expression study to check the impact of PPP on IGF1R and InsR levels. The expression was not affected by addition of PPP (data not shown).

### PPP inhibits proliferation, chemotaxis and signal transduction pathways activation

**Fig. 2:**
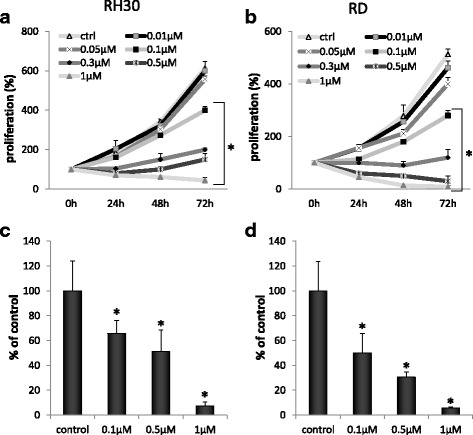
Effect of PPP on anchorage-dependent and anchorage-independent cell proliferation. RH30 (Panels **a** and **c**) and RD (Panels **b** and **d**) cells were treated with increasing doses of PPP (0–3 μM), and their proliferative capacity was measured in cultures in an anchorage-dependent plastic dishes (Panels **a** and **b**) and in anchorage-independent soft agar cultures (Panels **c** and **d**). Numerical values of proliferation are presented in relation to the number of cells at the beginning of the experiment (0 h time) that equaled 100%. Combined data from three independent experiments are shown. *p < 0.05

**Fig. 3:**
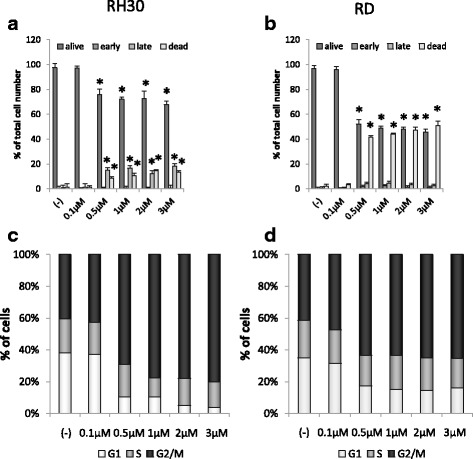
Cell cycle and apoptosis analysis of RMS cells exposed to PPP. RMS cells were treated with a broad range of PPP (0–3 μM) and apoptosis was evaluated by Annexin-V binding assay (Panels **a** and **b**) and cell cycle analysis was assayed with the use of Vybrant DyeCycle Orange Stain (Panels **c** and **d**). Higher (>0.5 μM) doses of PPP lead to accumulation of apoptotic cells. PPP inhibits cell proliferation in G2/M phase and decreases the number of cells in G1 phase. Data were collected by flow cytometry and analyzed by ModFit software. Cells were treated with PPP for 72 h. Panel **a** and **c** - RH30 cells; Panel **b** and **d** - RD cells. A representative analysis out of three independent experiments is shown. *p < 0.05

PPP is the potent growth inhibitor in multiple cancers [20–23]. In our study PPP effectively inhibited human Rhabdomyosarcoma cell proliferation in anchorage dependent assay on plastic dishes (Fig. 2 Panels a and b). Proliferation potential decreased in dose-dependent manner and the effects of growth arrest were significant after 72 h of treatment. Effective, subtoxic concentration was estimated to be 0.1 μM for both RH30 cells and RD treated for 72 h. Similarly, PPP inhibited colony formation on soft agar in an anchorage independent assay (Fig. 2 Panels c and d). Next, we tested the effect of PPP on cell viability/apoptosis and cell cycle (Fig. 3). In this experiment, flow cytometric measurement (FACS-based PI staining and Annexin V binding assay) was used to quantify the extent of apoptosis in the total cell population. 24-h exposure to PPP caused a dose-dependent decrease in number of alive cells and increase in the percentage of late apoptotic cells (FITC+, PI+) beginning from 0.5 μM and higher doses of PPP ≥ 2 μM, lead to massive apoptosis and cell death (Fig. 3 Panels a and b).

Furthermore, we noted that PPP caused cell cycle arrest in G2/M phase as shown by FACS-based time-lapse monitoring (Fig. 3 Panels c and d), and greatly decreases number of cells in G1 phase. This effect could not be reversed by addition of exogenous IGF1, IGF2 or insulin (data not shown).

**Fig. 4:**
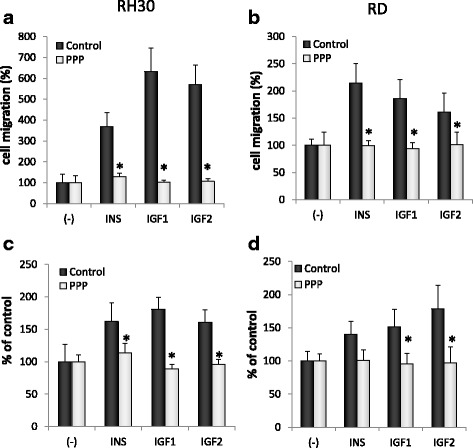
Chemotaxis and adhesion of RMS cells exposed to subtoxic dose of PPP in response to INS, IGF1, and IGF2 stimulation. RH30 (Panel **a** and **c**) and RD (Panel **b** and **d**) cells were pretreated for 30 min with 0.1 μM PPP and then stimulated with INS (10 ng/mL), IGF1 (100 ng/mL), or IGF2 (100 ng/mL) with 0.1 μM PPP added. Control cells were stimulated with INS, IGF1 and IGF2 only. In panels **a** and **b** subtoxic dose of PPP very efficiently blocked chemotaxis and in panel **c** and **d** the adhesion to fibronectin was inhibited. All experiments were repeated three times with similar results. *p < 0.05

In the following set of in vitro experiments we tested the effect of PPP on metastatic potential of RMS cells. We employed a subtoxic dose of PPP (0.1 μM) and a modified Boyden chamber chemotaxis assay. Two tested cell lines, RH30 and RD, exhibited strong chemotactic response towards insulin and insulin-like growth factor gradients (IGF1: 100 ng/mL, IGF2: 100 ng/mL, insulin: 10 ng/mL). At the same time a subtoxic dose of PPP very effectively blocked this response (Fig. 4 Panel a and b). To investigate the effects of PPP on tumor cell adhesion we used dishes coated with fibronectin. Similarly, cells pretreated with 0.1 μM PPP, prior to stimulation by IGF1, IGF2 or insulin, characterized with decreased adhesive potential when compared to controls (Fig. 4 panels c and d). What is interesting, in both assays – chemotaxis and adhesion, we noted that PPP effectively blocks insulin triggered responses.

**Fig. 5:**
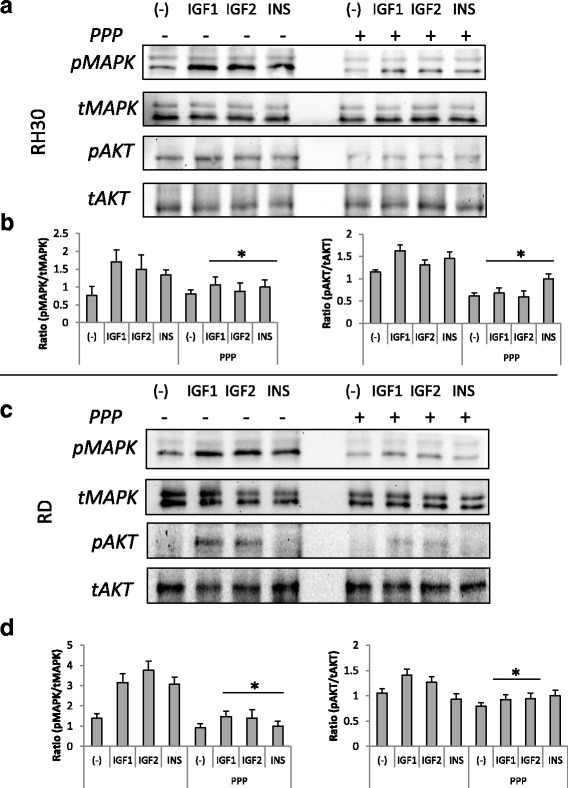
Effect of PPP treatment on INS, IGF1, and IGF2 signaling in RMS cells. RH30 (panel **a**) and RD (panel **c**) cells, untreated or PPP-treated (0.1 μM) for 72 h, were stimulated by INS (10 ng/mL), IGF1 (100 ng/ml), or IGF2 (100 ng/mL) for 5 min, and phosphorylation was assessed by western blotting. The experiment was repeated three times with similar results. Panels **b** and **d** show Western blot quantitation, expressed as a ratio of phosphorylated protein to total. *p < 0.05

Even though the exact mechanism of PPP action is not fully known, we wanted to check whether PPP decreases phosphorylation of kinases involved in cell proliferation and migration. RH30 and RD cells were incubated with PPP for 30 min and then stimulated with IGF1 (100 ng/mL), IGF2 (100 ng/mL) and insulin (10 ng/mL) for 5 min, next cell lysates where blotted against anti p-MAPK and anti p-AKT (p-S472) antibodies. Our results show that IGF1, IGF2 and insulin-induced phosphorylation is significantly decreased by PPP (Fig. 5).

### PPP increase rhabdomyosarcoma sensitivity to chemotherapy

**Fig. 6:**
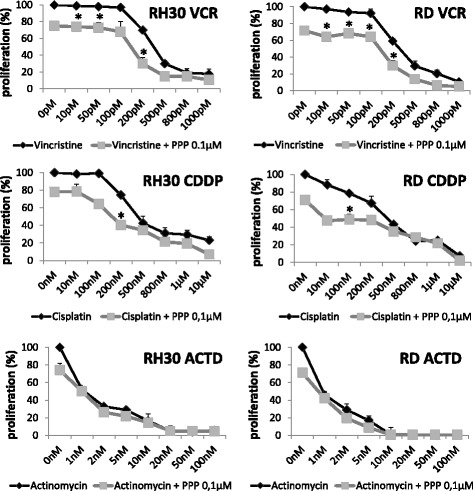
Concomitant treatment of RMS with chemotherapeutics (vincrsitine, actinomycin D and cisplatin) and subtoxic concentration of PPP (0.1 μM). RH30 and RD cells were simultaneously treated with indicated doses of chemotherapeutics and proliferation potential was assayed. (VCR – vincristine, CDDP – cisplatin, ACTD – actinomycin D). All experiments were repeated three times with similar results. *p < 0.05

Rhabdomyosarcoma treatment involves usage of a combination of chemotherapeutics. In our study we used three drugs: vincristine, actinomycin-D and cisplatin that are commonly used in cancer treatment [26, 27]. The experiments were aimed to determine the combined effects of chemotherapeutics and PPP on RMS proliferation. RH30 and RD cells were treated with rising doses of the drugs and a subtoxic dose of PPP (0.1 μM) for 72 h. In order to assess the type of drug-drug interaction we used fractional product method of Webb. We noted additive effects when vincristine and cisplatin was used concomitantly with PPP and antagonism in the case of actinomycin-D. Lower doses of chemotherapeutics were needed to obtain similar growth inhibition when additional PPP was used in subtoxic dose (Fig. 6).

### Xenotransplanted tumor growth is inhibited by PPP

**Fig. 7:**
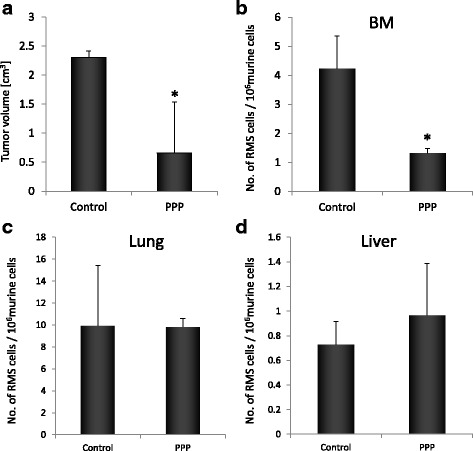
PPP inhibits tumor growth in vivo and metastasis. Panel **a**: Tumor formation by RH30 cells inoculated into the hind limb muscles of SCID/Beige inbred mice. Six weeks later, mice were sacrificed and femora were harvested to evaluate the size of the growing tumor. Five mice formed control group and five were treated with PPP (40 mg/kg/24 h) After two weeks from inoculation we started intrapertitoneal injection of PPP and vehicle alone (50 μl DMSO). Panel **b**, **c** and **d**: the human RMS cells were detected by RQ-PCR in the bone marrows, lungs and livers harvested from mice inoculated with RH30 (controls and PPP-treated). *p < 0.05

Finally, we employed a mouse xenotransplantation model. In the experiment we performed intramuscular injections of RH30 (ARMS) cells to SCID-beige mice and further divided them into two groups (control and PPP treated). A total of 10 SCID/beige mice were injected with RH30 cells (intramuscular, 6 × 10^6^ cells per leg). After two weeks from inoculation we started intraperitoneal injection of PPP (40 mg/kg/24 h) and vehicle alone (50 μl DMSO). We noted, that PPP-injected mice grew significantly smaller tumours as compared to controls (Fig. 7a). What is more we collected bone marrow, lungs and liver in order to estimate RMS cells seeding efficiency to these organs. DNA was isolated and using real-time RT-PCR we amplified human α-satellite sequences and murine β-actin. We found that mouse bearing RMS tumours that were treated with PPP exhibited 4 times lower amount of human cancer cells infiltrating bone marrow controls (Fig. 7b). Seeding efficiency to lungs and liver was not affected by PPP treatment. What is also very important, we did not notice significant side effects of PPP treatment, however mice injected with PPP had lower body mass (Additional file 1).

## Discussion

Oncogenic signalling through IGF1R tyrosine kinase has become a major focus of cancer research. IGF1R is considered a very important element in the development of childhood sarcomas. Cancers of the breast, colon, prostate, lungs or Ewing sarcoma belong to malignancies that show high expression of IGF1R [13–18, 28]. Increased expression of IGF1R is also characteristic for rhabdomyosarcomas. In work by Makawita et al., 93% of the examined RMS (both ARMS and ERMS) specimens showed high expression of IGF1 receptors. Similarly, 72–61% of therapy-naive biopsies stained positive for IGF1R (ARMS and ERMS respectively) [9]. In our study we evaluated and confirmed the high IGF1R expression on the surface of RMS cells from RH30 and RD cell lines that are characteristic of ARMS and ERMS subtypes respectively (Fig. 1 and [24]).

As the insulin-like growth factor (IGF) system is generally considered as a therapeutic target in multiple cancers, a lot of attempts have been made to target this pathway. Several strategies have been applied, including monoclonal antibodies and siRNA. These strategies, even those that prove to be very effective in in vitro models, are very difficult to apply in clinics and have shown some unexpected toxicity [28]. Thus, small molecule inhibitors may serve as potent alternatives in anti-IGF1R therapy. PPP, a member of the cyclolignan family, specifically blocks phosphorylation of the residue Tyr1136 in the activation loop of IGF1R kinase [21]. Interestingly, PPP can cause the complete regression of various types of human solid malignancies (prostate cancer,breast cancer, malignant melanomas and Ewing’s sarcomas) in animal models [21–24, 26, 29–32]. PPP (under the name AXL1717) is currently being investigated in clinical trials and the results presented to date suggest that it has at least some useful clinical activity in non-small lung carcinomas and exhibits only relatively low toxicity [33–35].

In our work, we employed several in vitro and in vivo techniques to assess the role of the IGF1R inhibitor PPP in human RMS. It was previously shown that PPP can be efficient as a growth inhibitor in murine alveolar and embryonal subtypes of RMS, with IC_50_ values of 150 and 200 nM respectively [36]. In our study, we noted that PPP is very effective in inhibiting proliferation of human RMS cells, both RH30 and RD with IC50 of around 0.1 μM (Fig. 2). RD cells were more sensitive and responded better to lower doses of the inhibitor. This is common for other IGF1R-positive tumour cells where PPP induced apoptosis and reduced cell survival, with IC_50_ values in the range of 0.05–0.5 μM [21–23].

Next, PPP was shown to halt cell cycling in RMS cells during the G2/M phase, which was previously reported in multiple myelomas [37] and other cancers [38]. Our results, presented in Fig. 2, indicate that PPP is an effective proliferation inhibitor and, in doses not exceeding 0.5 μM, apoptosis is not significant. Subsequently, we checked the effect of PPP on cell migration. It is known that both insulin-like growth factors and insulin are potent chemotactic agents and that they can stimulate RMS migration [6, 24, 25, 39]. Thus, we wanted to check whether PPP, except for growth inhibition, also attenuates cell migration. We used an in vitro chemotaxis assay with Transwell plates and applied subtoxic doses of PPP (0.1 µM). We observed that blockage of IGF1R does indeed stop cell migration. Similar results were obtained using a fibronectin assay and a MAPK/Akt phosphorylation assay. In the latter assay, cells treated with PPP one hour prior to stimulation with IGF1, IGF2 and insulin exhibited reduced signaling, as visualised by Western blot. Previously, PPP was shown to decrease the phosphorylation of IGF1R downstream pathways, MAPK and Akt in lung cancer [33], Ewing sarcomas [40] and glioblastomas [32]. Therefore, blockage of the MAPK signalling pathway inhibits cell migration and phosphorylated Akt works as an inhibitor of apoptotic proteins, thereby playing a crucial role in the growth of cancer cells [20].

Surprisingly, insulin triggered responses (chemotaxis, adhesion, MAPK or Akt phosphorylation) were blocked by PPP. PPP is characterised as very specific to IGF1R and has been previously shown not to interfere with insulin receptors [22]. One explanation for this phenomenon may be the existence of hybrid IR/IGF1R receptors that are sensitive to IGF1R inhibitors. This is a common situation in cancer, where cells show high expression of two receptors at the same time [41]. What is more, in our study we found that neither IGF1, IGF2 or insulin could overcome PPP inhibition in a proliferation assay. Similarly, growth of multiple myeloma cells treated with PPP could not be restored by insulin [37].

In the next step, we showed that PPP treatment increases sensitivity of rhabdomyosarcoma cells to classical chemotherapy treatment. Similar to the results obtained by Scotlandi et al. in musculoskeletal sarcoma cells. TC-71 cells were treated with IGF1R kinase inhibitor NVP-AEW541 [42], we noted that the subtoxic dose of PPP potentiated sensitivity to chemotherapeutic agents. In our study we noted that concomitant application of PPP and vincristine or cisplatin showed additive inhibitory effect on RMS cell survival. However, there was antagonistic response when actinomycin D was used. Possible explanation is ability of actinomycin D to stabilize the IGF1R mRNA transcript, increasing its expression [43] and thus counteracting blockage of IGF1R activity mediated by PPP.

Finally, for the first time, to our knowledge, we showed potent growth inhibition of human RMS tumours grown in mice by PPP. Consistent to previous studies, we found that PPP inhibited xenografted tumour growth and control in mice grown tumours, which were around 5 times bigger than PPP-treated tumours. What is more, PPP decreased the seeding efficiency of RMS cells to bone marrow, which is frequent site of metastasis in this type of cancer [6–12]. IGFs produced in bone marrow microenvironment may act as both chemotactic and pro-survival factors for the cancer cells [44, 45]. What is more, it was shown that bone marrow-derived MSCs are capable of transforming into cancer-associated fibroblasts (CAFs) within the primary tumor, and release IGF1 and CXCL12 creating initiation step of bone metastasis reviewed in [46]. Taken together with the fact that RMS cells highly express IGFs, blockage of IGF1R is a rational step in decreasing metastatic potential of Rhabomyosarcoma. PPP showed no obvious toxicity in mice, further supporting the potential safety and efficiency of PPP in the treatment of human cancer.

IGF1R together with IGF2 may form a short, very active, autocrine loop that is greatly responsible for tumour genesis, as well as intensive proliferation and progression of RMS [6]. Recently, we have shown that down-regulation of IGF2 by demethylating agents, such as 5′-Azacitidine, may disrupt this autocrine loop [24]. Interestingly, we have also shown that reactivation of H19 expression increases expression of microRNA 675. The miRNA has binding sites both for IGF1 and insulin receptors and decreases the expression of both receptors. Therefore, we may speculate that epigenetic demethylating drugs, such as AzaC, together with PPP may form an efficient tandem for blocking the IGF1R-IGF2 signalling pathway.

## Conclusions

In our study we used potent IGF1R inhibitor – PPP. In a series of in vitro experiments PPP efficiently attenuated IGF1 and IGF2-triggered responses. Moreover, PPP inhibited insulin mediated actions, supposedly through existing receptor heterodimers (IGF1R-InsR). In xenotransplantation studies PPP proved to significantly decrease tumour volume without toxic side effects. This study has clinical significance because Picropodophyllin is currently being used in clinical trials in other solid cancer and PPP may complement presently used chemotherapy.

## Additional file

Additional file 1: Xenotransplanted mice body mass comparison. Average body mass of mice inoculated with RMS cells (controls) and PPP-treated xenotransplanted mice. (PPTX 36 kb)

## Abbreviations

AKT: Serine/threonine-protein kinases
ARMS: Alveolar Rhabdomyosarcoma
AzaC: Azacitidine
DMSO: Dimethyl sulfoxide
ERMS: Embryonal Rhabdomyosarcoma
FACS: Fluorescence-activated cell sorting
FBS: Fetal bovine serum
IGF1: Insulin-like growth factor 1
IGF1R: Insulin like growth factor 1 receptor
IGF2: Insulin-like growth factor 2
IGF2R: Insulin like growth factor 2 receptor
Ins: Insulin
InsR: Insulin receptor
MAPK: Mitogen-activated protein kinase
PBS: Phosphate Buffer Saline
PPP: Picropodophyllin
PPT: Podophyllotoxin
RMS: Rhabdomyosarcoma
RPMI: Roswell Park Memorial Institute medium 1640
RT-PCR: Real-time quantitative reverse transcription PCR
SDS-PAGE: Sodium dodecyl sulfate-polyacrylamide gel electrophoresis
SEM: Standard error of the mean
