# Supplementary figures and images for: Picropodophyllin (PPP) is a potent rhabdomyosarcoma growth inhibitor both in vitro and in vivo
Source: BMC Cancer. 2017 Aug 9;17:532. doi: 10.1186/s12885-017-3495-y (PMC5550998; doi:10.1186/s12885-017-3495-y)

## Slide 1
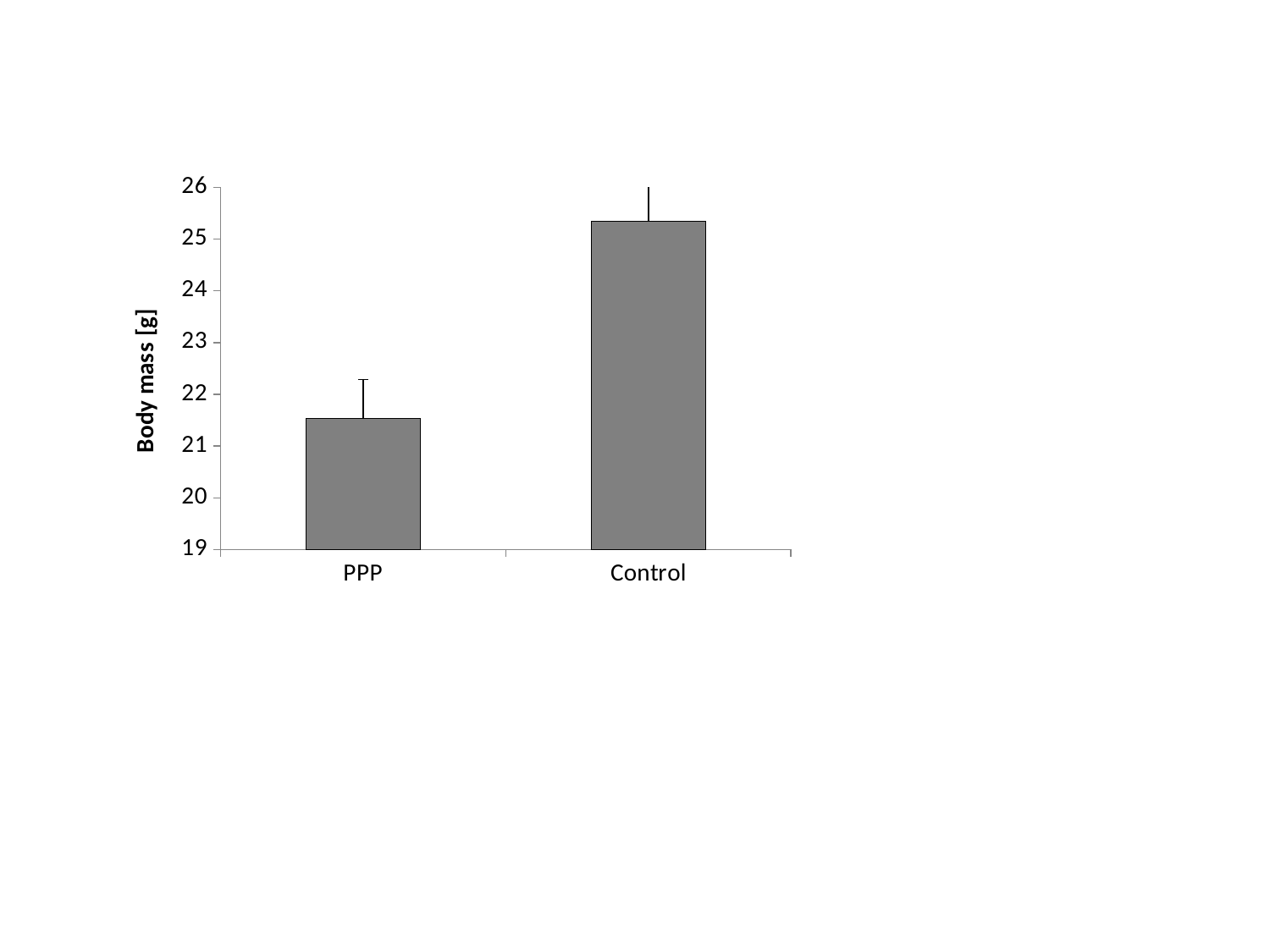

### Chart
| Category | |
|---|---|
| PPP | 21.53333333333333 |
| Control | 25.349999999999998 |

Supplement: Additional file 1: — Xenotransplanted mice body mass comparison. Average body mass of mice inoculated with RMS cells (controls) and PPP-treated xenotransplanted mice. (PPTX 36 kb) [file 12885_2017_3495_MOESM1_ESM.pptx]
